# Tensor Decomposition Analysis of Longitudinal EEG Signals Reveals Differential Oscillatory Dynamics in Eyes-Closed and Eyes-Open Motor Imagery BCI: A Case Report

**DOI:** 10.3390/brainsci13071013

**Published:** 2023-06-30

**Authors:** Saman Seifpour, Alexander Šatka

**Affiliations:** 1 RIKEN Center for Brain Science, Wako, Saitama 351-0198, Japan; 2 Institute of Measurement Science, Slovak Academy of Sciences, Dubravska cesta 9, 84104 Bratislava, Slovakia

**Keywords:** Brain-computer interfaces (BCIs), motor imagery, EEG oscillations, sensorimotor rhythms, eyes-open, eyes-closed, tensor decomposition, PARAFAC

## Abstract

Functional dissociation of brain neural activity induced by opening or closing the eyes has been well established. However, how the temporal dynamics of the underlying neuronal modulations differ between these eye conditions during movement-related behaviours is less known. Using a robotic-assisted motor imagery brain-computer interface (MI BCI), we measured neural activity over the motor regions with electroencephalography (EEG) in a stroke survivor during his longitudinal rehabilitation training. We investigated lateralized oscillatory sensorimotor rhythm modulations while the patient imagined moving his hemiplegic hand with closed and open eyes to control an external robotic splint. In order to precisely identify the main profiles of neural activation affected by MI with eyes-open (MIEO) and eyes-closed (MIEC), a data-driven approach based on parallel factor analysis (PARAFAC) tensor decomposition was employed. Using the proposed framework, a set of narrow-band, subject-specific sensorimotor rhythms was identified; each of them had its own spatial and time signature. When MIEC trials were compared with MIEO trials, three key narrow-band rhythms whose peak frequencies centred at ∼8.0 Hz, ∼11.5 Hz, and ∼15.5 Hz, were identified with differently modulated oscillatory dynamics during movement preparation, initiation, and completion time frames. Furthermore, we observed that lower and higher sensorimotor oscillations represent different functional mechanisms within the MI paradigm, reinforcing the hypothesis that rhythmic activity in the human sensorimotor system is dissociated. Leveraging PARAFAC, this study achieves remarkable precision in estimating latent sensorimotor neural substrates, aiding the investigation of the specific functional mechanisms involved in the MI process.

## 1. Introduction

Brain-computer interfaces (BCIs) have emerged as a reliable assistive and rehabilitative technology with proven clinical efficacy for cognitive and motor skills enhancement in healthy subjects [1,2] and patients with motor disabilities resulting from focal lesions [3,4,5]. Preliminary evidence suggests that stroke patients’ ability to perform movement-related tasks is similar to healthy subjects [6,7], in which specific brain regions are engaged resulting in a decrease or increase in electroencephalographic (EEG) activity (with respect to a baseline) known as event-related desynchronization or synchronization (ERD/ERS) [8]. This is an important finding providing a basis for the popularity of the motor imagery BCI (MI BCI) applications in functional recovery in post-stroke patients. Nevertheless, the dependence of the MI BCI performance on the patient’s concentration during MI execution is a challenging problem in this area [9,10,11,12,13,14].

On the other hand, there is a growing body of literature that suggests brain neural oscillations are strongly modulated by the subject’s eye conditions [15,16]. Alpha wave, as the primary and well-known EEG activity, has been historically evidenced as a dominating characteristic of EEG during resting eyes-closed (EC) condition suppressed in eyes-open (EO) condition [17]. Task-related alpha activation has been shown to mainly affect the occipital, parietal, and frontal brain regions during EC, whereas EO alpha modulations are particularly significant in the posterior areas [18,19]. Over the past two decades, neuroimaging studies utilizing functional magnetic resonance imaging (fMRI) have revealed more detailed information about the substantial differences in the activation patterns of brain functional and structural networks between resting EO and EC states [20,21,22,23,24]. These significant differences in spontaneous brain activity between resting EO and EC states have also been observed in the sensorimotor cortex [25], which coordinates movement-related activities such as active/passive movements or motor imagery/planning [26,27].

Although extensive research has been carried out on resting-state brain activation in EC and EO, scant attention has been paid to the influence of the eyes’ conditions on the modulations of the sensorimotor cortex during the performance of a movement-related task [28,29]. However, no single study exists that investigates this subject in the BCI context, where participants try to control a matched sensory feedback or an external robotic device by MI [29]. This aspect holds particular significance in clinical settings [30], as the act of opening or closing the subject/patient’s eyes can significantly impact BCI performance and the efficiency of rehabilitation.

Therefore, using a single-case longitudinal paradigm, this study attempts to shed light on neural processes underlying MI with EO and EC during BCI-based post-stroke rehabilitation using EEG. Due to the neuroplasticity characteristic that enables the reorganization of neural pathways in the brain [31], it is hypothesized that specific brain networks associated with motor function recovery can be activated after each MI BCI training session [32,33]. These networks not only have a specific spatial distribution (laterally distributed over the sensorimotor cortex [34,35]) but also have specific spectral signatures that may vary throughout the course of rehabilitation (different recording times) or experimental conditions (different eye conditions) [36,37]. This imposes many dimensions such as time, frequency, space, trials, and tasks on the recorded data. For such highly multidimensional data, standard matrix (a two-way tensor) factorization methods such as fast Fourier transform (FFT) or principal/independent component analysis (PCA/ICA) might fail to represent a comprehensive representation of the original multidimensional data structure [38]. Therefore, advanced signal processing methods based on tensor decomposition encompassing these additional dimensions should take higher priority.

In signal processing, the term *tensor* refers to an *N*-way or (multidimensional) array characterized by more than two modes. If multidimensional data follow a low-rank tensor approximation, they can be represented by higher-order tensors and compressed into multiple unique components with distinct modalities using tensor decomposition methods [39]. Parallel factor analysis (PARAFAC) [40] is one of the powerful *N*-way tensor decomposition approaches, and its promising applicability in modelling EEG data has been undertaken through a wide range of clinical [41,42], cognitive [43,44], and BCI [45,46,47] studies.

Accordingly, this study leverages PARAFAC to precisely analyze underlying neural mechanisms reflecting MI with eyes-open (MIEO) versus eyes-closed (MIEC), thereby providing deeper insights into differential oscillatory dynamics associated with MIEO and MIEC. Ultimately, these findings have the potential to refine and optimize rehabilitation protocols, presenting new possibilities for the design of personalized, effective approaches, tailored to post-stroke patients.

## 2. Material and Methods

### 2.1. Participant

It should be stressed that the raw data used in this study come from the existing dataset collected at the Institute of Measurement Science of the Slovak Academy of Sciences (IM SAS). The EEG data of a male subject (58-year-old), whose left frontotemporal to parietal brain regions were damaged due to an ischemic stroke, have been analyzed in this study. At the beginning of the rehabilitation course, clinical assessments using the Fugl-Meyer Assessment (FMA) [48], Modified Stroke Impact Scale (MSIS) [49], and Modified Ashworth Scale (MAS) [50] revealed a severe impairment in his right-hand upper limb. Having been fully informed of the study, he then signed a written consent form. Note that although this manuscript builds on the data from our prior studies [46,47], the current study was conducted based on a different research question, and all analyses reported here are novel.

### 2.2. Motor Imagery Task

**Figure 1:**
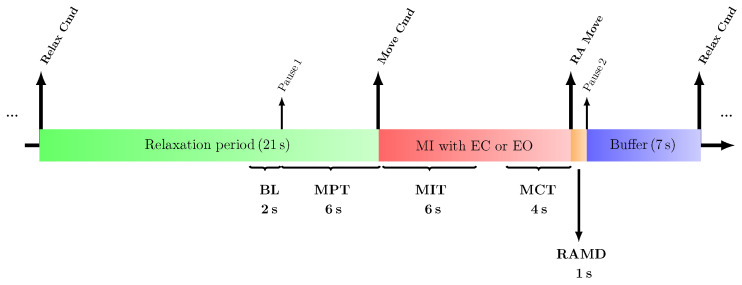
Schematic illustration of a successful MI trial (either with EC or EO) in which the patient was able to hit the robotic splint coupled with the designed BCI after 12 seconds. The duration of a robotic arm movement (RAMD; orange box) is one second. The interval between ending a trial (i.e., the MI period; red box) and starting a new trial (i.e., the Relaxation period; green box) is separated by implicit transition periods as a buffer zone (blue box). The considered baseline (BL), movement preparation (MPT), movement initiation (MIT), and movement completion (MCT) time frames are also indicated.

At each rehabilitation session, the participant sat down in a comfortable armchair while his right forearm was fixed to the robotic splint, and a trained technician recorded EEG data. Each training session began (pre-training) and ended (post-training) with two minutes of resting-state EEG recordings with closed eyes, followed by two minutes with open eyes. Between the two pre- and post-training blocks, the subject performed 20 trials of a movement imagination task using a robotic-assisted MI BCI, during which the conditions of MIEC and MIEO were alternating for every 10 trials. Each trial started with a 21-second relaxation period (the green box in Figure 1), signalled by a voice cue (*Relax* command). The second voice cue (*Move* command) was then indicated when the patient had to begin the MI task (the red box in Figure 1). The patient was allowed a maximum of 20 seconds to imagine moving his hemiplegic hand (without real movement). He was rewarded with a real robotic splint movement (the orange box in Figure 1) if he modulated the target signal appropriately. Otherwise, a subsequent trial would begin when the MI time frame ran out. OpenViBE (v2.0.1) software was used to implement this cue-based paradigm [51]. Previous to participation in this study, the patient also completed a course of rehabilitation using mirror-box therapy [41]. In the online signal processing step, the general frequency and spatial weights (see Section 3.2) of the patient in the range of 7.5 to 8.75 Hz (showed a clear correlation with passive movement or active movement [41]) obtained during mirror-box therapy were continuously projected to every two seconds (with a step size equal to 500 ms) of raw EEG spectrum to obtain a time score for each time window (see Section 2.5.4). The binary feedback of successful MI was given by triggering the motor-driven splint to move the right-handed wrist from $0^{\circ }$ to $40^{\circ }$, whereas the splint was immovable during unsuccessful MI trials. The threshold for hitting the robotic device was set between 15% and 20% of decreases in the time scores with respect to the relaxation period.

### 2.3. Data Acquisition

Continuous EEG data were recorded from A2, and 11 scalp electrodes covered the left (FC3/ C1/C3/C5/CP3) and right (FC4/C2/C4/C6/CP4) motor cortices as well as the left occipital lobe (O1), referenced to A1 using g.GAMMAcap (g.tec medical engineering GmbH). The ground electrode was placed at Fpz. The electrode arrangement was in accordance with the 10-5 EEG electrode placement system [52]. All impedances were below 10 kΩ at the start of the recording. Data were sampled at 512 Hz and band-pass filtered between 0.1–200 Hz in the g.USBAMP amplifier (g.tec medical engineering GmbH).

### 2.4. Data Pre-Processing

For pre-processing, EEG data were firstly down-sampled to 128 Hz. In the first step, a threshold-based automated algorithm implemented in BrainVision Analyzer 2 software (Brain Products GmbH) was employed. The artefact-inclusion criteria involved setting a maximum allowed voltage step of 50 μV/ms, a minimum allowed activity of 0.5 μV in intervals of 100 ms, and a maximum allowed voltage difference of 50 µV in intervals of 20 ms. If any of these criteria were met, the interval preceding and following the detected artefact by 150 ms was marked as bad, except for the third criterion where the interval was set to 50 ms. After the automatic artefact detection, the EEG data were visually inspected using the same software by an expert who manually marked additional periods with undetected artefacts. The artefact markers that were incorrectly assigned automatically, including the detection and removal of ocular artefacts, were also removed by the expert. More details regarding our pre-processing step have been presented in our previous studies [47]. After pre-processing, we retained on average (± SD) 95.6% (± 2.3%) and 84.2% (± 6.0%) of the EEG data recorded during MI with EC and MI with EO, respectively.

### 2.5. Tensor Analysis

#### 2.5.1. Tensor Equation

PARAFAC decomposes an *N*-way tensor $X\in R^{I_{1}\times I_{2}\times ...\times I_{N}}$ into *N* matrices:

(1) $$\begin{array}{c} \begin{array}{c} A^{\left(n\right)}=\left(a_{1}^{\left(n\right)},a_{2}^{\left(n\right)},...,a_{F}^{\left(n\right)}\right)\in R^{I_{n}\times F} \end{array} \end{array}$$

**Figure 2:**
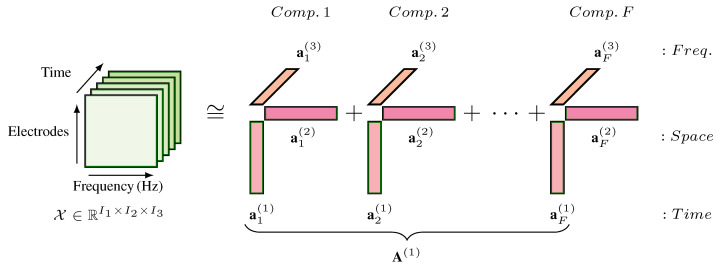
Schematic illustration of an *F*-component PARAFAC model of the three-dimensional tensor X indicated by time, space, and frequency. Each mode (dimension) comprises *F* vectors $a_{1}^{\left(n\right)}$, $a_{2}^{\left(n\right)}$, and $a_{F}^{\left(n\right)}$ forming the columns of matrix $A^{\left(n\right)}$. Each component comprises three vectors $a_{i}^{\left(1\right)}$, $a_{i}^{\left(2\right)}$, and $a_{i}^{\left(3\right)}$. The residual tensor E was omitted from the figure to simplify the model.

In the given tensor equations, the symbol $A^{\left(n\right)}$, n=1,...,N represents a collection of matrices $\left\{A^{\left(1\right)},A^{\left(2\right)},...,A^{\left(N\right)}\right\}$, where *N* is the number of modes in the tensor decomposition. Each matrix $A^{\left(n\right)}$ has dimensions $I_{n}\times F$, where $I_{n}$ represents the size of the $n^{\left(th\right)}$ mode and *F* represents the number of factors or latent components. The vector $a_{i}^{\left(n\right)}\in R^{I_{n}}$, i=1,...,F, denotes the *i*th column of $A^{\left(n\right)}$ (see Figure 2). This follows the formula below:

(2) $$\begin{array}{c} \begin{array}{c} X=G\times_{1}A^{\left(1\right)}\times_{2}A^{\left(2\right)}\times_{3}...\times_{N}A^{\left(N\right)}+E,\\ x_{i_{1},i_{2},...,i_{N}}=\sum_{f=1}^{F}g_{f}a_{i_{1}f}^{\left(1\right)}\;a_{i_{2}f}^{\left(2\right)}...a_{i_{N}f}^{\left(N\right)}+e_{i_{1}i_{2}...i_{N}},\\ i_{j}=1,2,...,I_{j}\;;\;j=1,2,...,N, \end{array} \end{array}$$

where $\times_{j}$, j=1,2,...,N denotes the tensor-matrix product in the *j*th mode and $G\in R^{F\times F\times ...\times F}$ known as core tensor term following an *N*-way super-diagonal configuration with non-zero elements only on the main diagonal. The tensor $E\in R^{I_{1}\times I_{2}\times ...\times I_{N}}$ represents an error term of the model.

#### 2.5.2. Tensor Construction

With the aim of detecting dominant motor-related EEG sensorimotor oscillations for each training day and for each eye condition (MIEC or MIEO), the collected EEG data were modelled separately by a 3-way (time × electrode × frequency) PARAFAC (see Figure 2). To this end, the artefact-free multi-channel EEG data recorded during MI BCI training were split into two-second sliding segments with an overlap of 500 ms. Then, for each segment and electrode, the EEG spectrum’s oscillatory components were estimated by irregular resampling auto-spectral analysis (IRASA) [53]. IRASA separates oscillatory components of electrophysiological data by subtracting the ubiquitous non-oscillatory part from the raw power spectra. Following our previous studies [46,47], the oscillatory spectrum was restricted to the frequency range of between 4 and 25 Hz and log-transformed. In the three-dimensional tensor, the number of two-second long epochs varied based on the recording session and MI condition, but the number of electrodes and selected frequencies were stable and equal to 10 (the O1 electrode was excluded), and 43 (4 to 25 Hz with a 0.5 Hz step), respectively. The tensor was then zero-centred across the first mode or dimension (time). Following the previous studies [41,46,47,54], additional constraints such as unimodality and non-negativity were imposed on the factor matrices to facilitate the neurophysiological interpretation of the results. Tensor analysis was performed using Matlab by the *APECSgui* toolbox [55], including the *N*-way toolbox [56].

#### 2.5.3. Number of Factors

According to *tripleC* method [57], the number of factors in our PARAFAC models for each neurorehabilitation session and each eye condition varied between 6 and 20. A semi-automatic approach, including non-parametric density-based cluster analysis using the DBScan clustering algorithm [58] followed by visual inspection, was then applied to all the extracted weights from all models according to our previous studies [46,47].

#### 2.5.4. Generating Atom-Specific Time Scores

For generating time scores, the dominant PARAFAC spatial and spectral weights of all available training sessions for each MIEC and MIEO condition were first averaged together to obtain general atoms. The general spatial and spectral atoms were then projected to the oscillatory EEG spectrum estimated for two-second long overlapping segments with a sliding step size of 7.8125 ms (= 1/128 Hz, i.e., one data point shift). In other words, the projection was performed for each EEG sample, resulting in the generation of a smooth numerical sequence called time score, in which each value of the time score represents the presence of a specific atom (in the EEG spectrum) and space at a given time. For each MIEO or MIEC condition, a distinct time score was obtained. It is worth clarifying that in this study, narrow-band EEG rhythms are termed *atoms* and how strongly each EEG atom is present over time is termed *time scores* [43,46,47].

### 2.6. Statistical Analysis

Statistical analysis was performed using Matlab (R2019b) [59]. We considered non-parametric, cluster-based permutation statistics for evaluating differential modulatory effects of atom-specific time scores induced by MIEC versus MIEO. This approach has been identified as a reliable way to increase the sensitivity of statistical testing and to control for multiple comparisons in neuroimaging data [60]. The cluster-based analysis was obtained by 10,000 permutations using a threshold of p<0.05 (a two-sided test). We also used a chi-square test of independence to test whether or not there was a statistically significant association between two eye conditions and behavioural performance, including MI duration (the time required for triggering the robotic splint) and MI success rate (the number of times that the robotic splint was triggered).

## 3. Results

### 3.1. Eyes Condition-Specific Non-Oscillatory and Oscillatory Activities

The raw spectrum, non-oscillatory, and oscillatory components were computed by IRASA for each channel, BCI training session, and MI condition. To this end, the MI data were segmented into four-second sliding windows with an overlap of 500 ms, resulting in a frequency resolution of 0.25 Hz. The number of analyzed segments per session per condition after data cleaning was: 860.4 ± 50.9 and 892.7 ± 52.0 (mean ± SD) for the MIEC and MIEO conditions, respectively.

**Figure 3:**
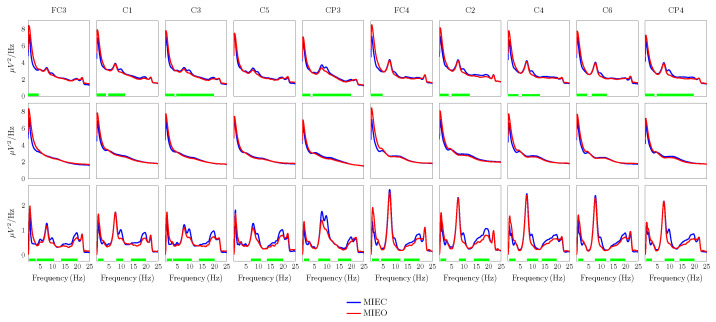
Raw power spectrum (*first row*), non-oscillatory part (*second row*), and oscillatory part (*third row*) of EEG data recorded during the MIEC (*blue*) and MIEO (*red*) conditions. These components were estimated by the irregular-resampling auto-spectral analysis (IRASA) and averaged over all available training sessions. Statistically significant clusters indicating the difference between the MIEC and MIEO EEG components are indicated by green bars along the x-axis.

Figure 3 shows the comparison of the EEG spectral characteristics of the EC and EO conditions across the investigated spectrum. The figure was obtained by averaging raw spectrum, non-oscillatory, and oscillatory parts for each eye condition over all available training days at each electrode location. Overall, we found that narrow-band differences in EEG power between the two conditions are more pronounced in the oscillatory part of the EEG spectrum (Figure 3, third row). These differences were identified by the cluster-based permutation statistics (using frequency as the clustering dimension), indicating that EO oscillatory power is significantly lower than power during the EC condition. These clusters were especially obvious around the peak frequency occurring at 7.5 Hz and in the frequency range between 14 Hz and 20 Hz. Meanwhile, such dissociated clusters, representing localized rhythmic activity within a narrow-band frequency range, were not recognisable in the raw spectrum. On the other hand, the significant clusters of the global reduction in slow EEG activities (less than 5 Hz) of EC compared with EO were detectable in the raw spectrum as well as the oscillatory component of the spectrum. In the frequency interval of 20-25 Hz, power (both raw spectrum and oscillatory part) in the EC condition was lower than for EO, but the difference between the two conditions was insignificant. Furthermore, there were no significant differences in non-oscillatory EEG power in the EO versus the EC MI conditions across all electrodes, as evidenced by cluster-based permutation statistics.

### 3.2. Eyes Condition-Specific Sensorimotor Oscillatory Activity

**Figure 4:**
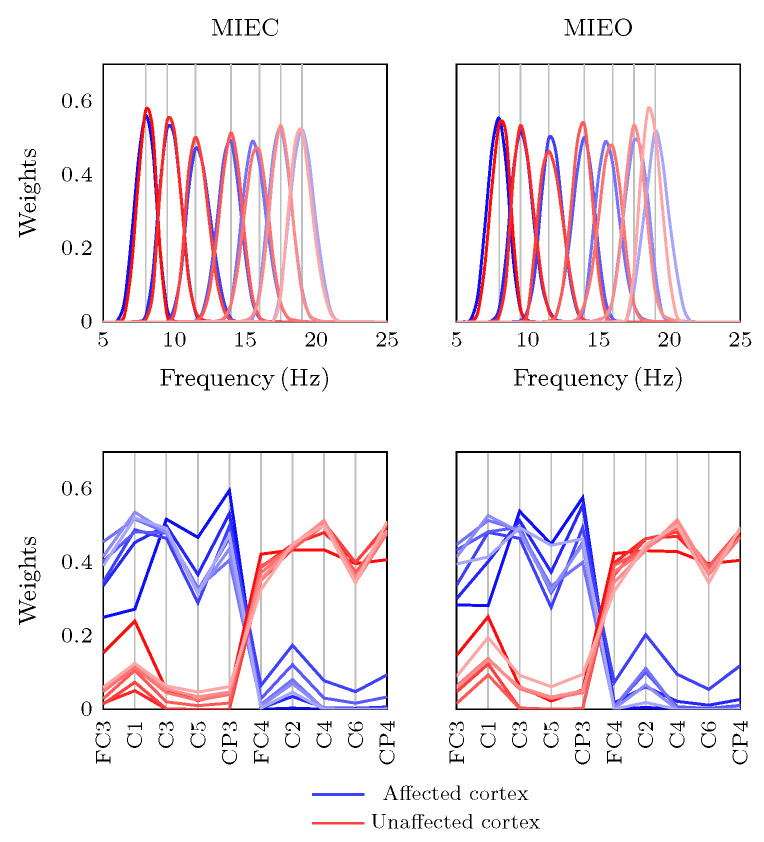
General frequency (*first row*) and spatial (*second row*) PARAFAC atoms of multichannel EEG data recorded during MIEC (*left column*) and MIEO (*right column*). The *blue* and *red* spectra depict the lateralized spatial weights of the corresponding narrow-band EEG oscillatory rhythms over the affected and unaffected cortices, respectively. The peak of each narrow-band EEG oscillatory rhythm and the spatial weight of each EEG electrode are shown in grey vertical lines.

Using the three-way PARAFAC, we identified seven dominant general frequency atoms with a clear unimodal structure, which were consistent and relatively repeatable across training days. These atoms closely corresponded to the underlying lateralized spatial signatures presenting cortical sensorimotor EEG oscillations over the left and right hemispheres. As shown in Figure 4, for each MIEC and MIEO condition, the model identified almost the same EEG oscillatory rhythms with peak frequencies at 8.0, 9.5, 11.5, 14.0, 15.5, 17.5, and 19.5 Hz. The negligible difference in the central peak frequencies can be interpreted as numerical issues in PARAFAC solutions. The grey vertical lines in Figure 4 show the central frequency of the identified motor-related sources (first row), and corresponding spatial weights of each EEG electrode (second row).

### 3.3. Dynamic Characteristics of MIEC Versus MIEO during Movement Preparation and Movement Initiation Time Frames

The first approach to investigating the modulatory difference of sensorimotor rhythms underlying MIEO and MIEC is to compare the evoked oscillatory activity in movement preparation and movement initiation time frames. In this first step, for calculating time score averages, we considered all MI trials recorded throughout all rehabilitation sessions, regardless of whether they were successful or unsuccessful (whereby the patient was *unable* to hit the robotic splint). We believed that even in the unsuccessful trials, the patient was engaged in the MI task, but his efforts were not enough to get the defined threshold and control the robotic splint. Meng et al. has also demonstrated this case [61].

In order to do so, the trial time scores for all available days were averaged separately over the ipsilateral (right) and contralateral (left) sensorimotor cortices to smooth the data and gain a maximal signal-to-noise ratio. Here, we empirically selected the z-score method among various methods that exist for baseline correction [62]. Firstly, we calculated the average time score values of each MI effort of each trial within the defined baseline interval. This average was then subtracted from the considered time interval of the time scores. The result was divided by the standard deviation baseline time score values.

**Figure 5:**
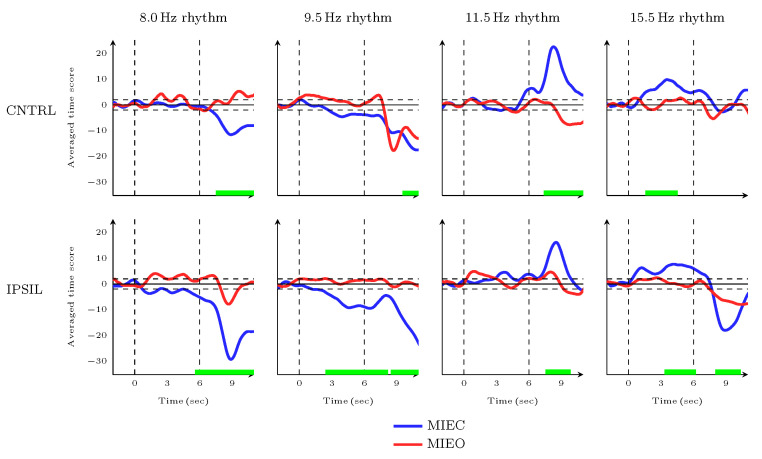
Temporal dynamics of the baseline-corrected 8.0 Hz (*first column*), 9.5 Hz (*second column*), 11.5 Hz (*third column*), 15.5 Hz (*fourth column*) oscillatory rhythms during the time frames of movement preparation (MPT) and movement initiation (MIT). The time scores were detected over the sensorimotor cortex contralateral (CNTRL; *first row*) and ipsilateral (IPSIL; *second row*) to the imagined hand during MIEC (*blue*) and MIEO (*red*) conditions and averaged over all available training sessions. The vertical dashed lines show the onset of *Relax* and *Move* voice commands, respectively. The horizontal dashed lines show the level of significance at 5%. The green bars along the x-axes represent the time clusters in which the differences between neural dynamics of the two MI conditions are significant.

**Figure A1:**
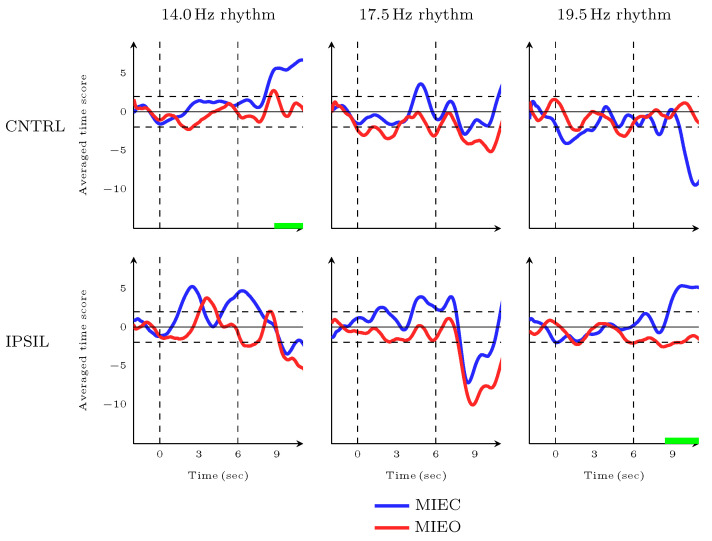
Temporal dynamics of the baseline-corrected 14.0 Hz (*first column*), 17.5 Hz (*second column*), 19.5 Hz (*third column*) oscillatory rhythm’ modulations during the time frames of movement preparation (MPT) and movement initiation (MIT). The time scores were detected over the sensorimotor cortex contralateral (CNTRL; *first row*) and ipsilateral (IPSIL; *second row*) to the imagined hand during MIEC (*blue*) and MIEO (*red*) conditions and averaged over all available training sessions. The vertical dashed lines show the onset of *Relax* and *Move* voice commands, respectively. The horizontal dashed lines show the level of significance at 5%. The green bars along the x-axes represent the time clusters in which the differences between neural dynamics of the two MI conditions are significant.

For this purpose, the ipsi- and contralateral time scores of each identified frequency atom representing narrow-band EEG oscillatory rhythms were segmented into 14-second epochs. In other words, each epoch included a time frame of 2 seconds before the movement preparation onset (i.e, BL in Figure 1) considered as a baseline, 6 seconds movement preparation time frame (i.e, MPT in Figure 1), and 6 seconds after the sound cue presentation (i.e, MIT in Figure 1) considered as the movement initiation time frame. The results of the comparative analysis are presented in Figure 5 and Figure A1.

In the movement preparation phase, there was a strong enhancement in 15.5 Hz atom over both ipsi- and contralateral sensorimotor cortices for MIEC compared to MIEO. In contrast, the baseline-corrected time score of 9.5 Hz atom ipsilateral to the hand used for imagery showed a significant decrease for MIEC compared with the MIEO during the movement preparation phase. Applying cluster-based permutation statistics (using time as the clustering dimension) showed significant clusters between 4550 and 1500 ms (for contralateral 15.5 Hz atom, p=0.014), between 2700 and 0 ms (for ipsilateral 15.5 Hz atom, p=0.002), and between 3450 and 0 ms (for ipsilateral 9.5 Hz atom, p<0.001) within the time frame of the movement preparation (i.e -6000 ms to *Move Command* presentation or MI onset).

When the patient started to imagine moving his affected hand with closed eyes, the magnitude time score suppression of 8.5 Hz and 9.5 Hz atoms over both ipsil- and contralateral sensorimotor cortices and 15.5 Hz atom over the ipsilateral cortex was significantly higher compared with the MIEO trials. The observed differences were confirmed by the cluster-based permutation statistics (using time as the clustering dimension), where the statistically significant clusters for decreases in oscillatory rhythm time scores were identified in the 8.0 Hz (ipsilateral: between 6000 and 12,000 ms p<0.001; contralateral: between 7500 and 12,000 ms p<0.001), 9.5 Hz (ipsilateral: between 6000 and 8100 ms, and between 8500 and 12,000 ms p<0.001; contralateral: between 9500 and 12,000 ms p<0.001), and 15.5 Hz (ipsilateral: between 8000 and 10,350 ms; p=0.002) atoms after stimulus presentation within the time frame of the movement initiation. Furthermore, 11.5, 14.0, and 19.5 Hz oscillatory atoms showed stronger increases in their time score activities for the MIEC condition over the ipsilateral (for 11.5 Hz and 19.5 Hz) and contralateral (for 11.5 Hz and 14.0 Hz) sensorimotor cortices compared with the MIEO trials. Clusters of significant differences between MIEC and MIEO conditions were identified by permutation statistics within the time frame of the movement initiation for 11.5 Hz (ipsilateral: between 7550 and 9950 ms p=0.007; contralateral: between 7400 and 10750 ms p<0.001), 14.5 Hz (contralateral: between 8800 and 12,000 ms p=0.001), and 19.5 Hz (ipsilateral: between 8850 and 12000 ms p<0.001) atoms.

### 3.4. Dynamic Characteristics of MIEC Versus MIEO during Movement Completion Time Frame

In the second approach, to investigate time-locked temporal dynamics underlying the MI process with EC and EO, atom-specific time scores were studied within 4-second time frames (i.e, MCT in Figure 1) before triggering the robotic arm, relative to a baseline interval. The same baseline as outlined in the previous Section 3.3, was selected 2 seconds before the movement preparation onset (i.e., BL in Figure 1). Accordingly, only successful trials in which the patient was *able* to hit the robotic splint were included in calculating time score averages.

**Figure 6:**
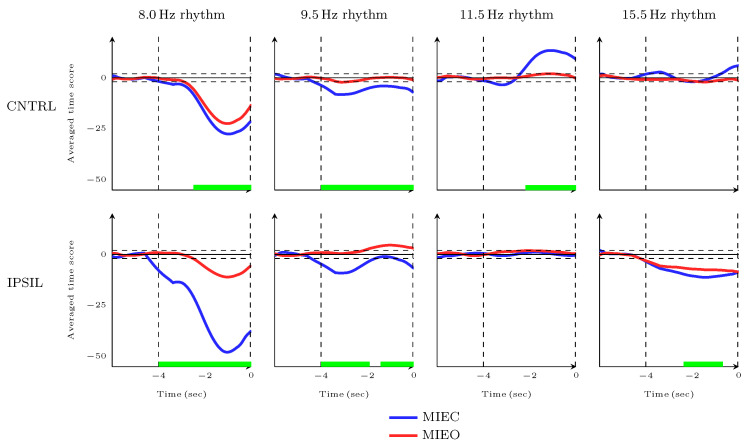
Temporal dynamics of the baseline-corrected 8.0 Hz (*first column*), 9.5 Hz (*second column*), 11.5 Hz (*third column*), 15.5 Hz (*fourth column*) oscillatory rhythm’ modulations during the time frame of movement completion (MCT). The time scores were detected over the sensorimotor cortex contralateral (CNTRL; *first row*) and ipsilateral (IPSL; *second row*) to the imagined hand during MIEC (*blue*) and MIEO (*red*) conditions and averaged over all available training sessions. The vertical dashed lines show the time-locked and robotic splint movement onsets (i.e. movement completion). The horizontal dashed lines show the level of significance at 5%. The green bars along the x-axes represent the time clusters in which the differences between neural dynamics of the two MI conditions are significant.

**Figure A2:**
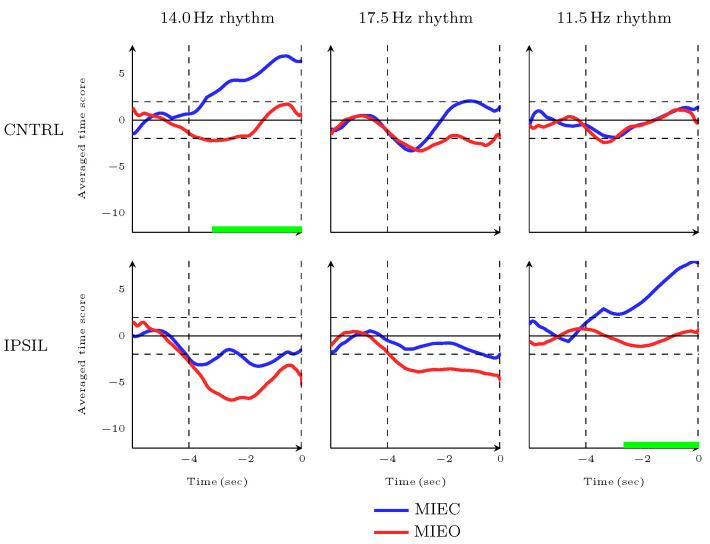
Temporal dynamics of the baseline-corrected 14.0 Hz (*first column*), 17.5 Hz (*second column*), 19.5 Hz (*third column*) oscillatory rhythm’ modulations during the time frame of movement completion (MCT). The time scores were detected over the sensorimotor cortex contralateral (CNTRL; *first row*) and ipsilateral (IPSIL; *second row*) to the imagined hand during MIEC (*blue*) and MIEO (*red*) conditions and averaged over all available training sessions. The vertical dashed lines show the time-locked and robotic splint movement onsets (i.e. movement completion). The horizontal dashed lines show the level of significance at 5%. The green bars along the x-axes represent the time clusters in which the differences between neural dynamics of the two MI conditions are significant.

As can be seen in Figure 6 and Figure A2, the baseline-corrected (z-score) time scores of MIEC significantly decreased in 9.5 Hz oscillatory atom but increased in 11.5 Hz and 14.0 Hz oscillatory atoms over the sensorimotor cortex contralateral to the hand used for imagery, as compared with MIEO. Furthermore, when comparing MIEC to MIEO, the suppression of bilateral 8.0 Hz and ipsilateral 15.5 Hz oscillatory atoms was the most pronounced over the sensorimotor network. Cluster-based permutation statistics (using time as the clustering dimension) revealed that the decreases in oscillatory power occurred in the ipsilateral 8.0 Hz atom 4000 ms (p<0.001), contralateral 8.0 Hz atom 2450 ms (p<0.001), and contralateral 9.5 Hz atom 4000 ms (p<0.001) before robotic arm movement within the defined time frame for movement completion. In addition, significant clusters of enhancement in contralateral 11.5 Hz, contralateral 14.0 Hz, and ipsilateral 19.5 Hz oscillatory atoms were detected by permutation statistics in 2150 ms (for 11.5 Hz atom, p=0.002), 3150 ms (for 14.0 Hz atom, p=0.001), and 2650 ms (for 19.5 Hz atom, p=0.001) before robotic arm movement. Furthermore, significant clusters of decreases in ipsilateral 9.5 Hz oscillatory atom between 4000 and 1900 ms (p=0.005) and between 1400 ms to robotic splint movement onset (p=0.040) as well as ipsilateral 15.5 Hz oscillatory atom between 2350 to 650 ms (p=0.034) were identified by the cluster-based permutation statistics.

### 3.5. Imagery Task Performance in Eyes-Closed Versus Eyes-Open Conditions

**Figure 7:**
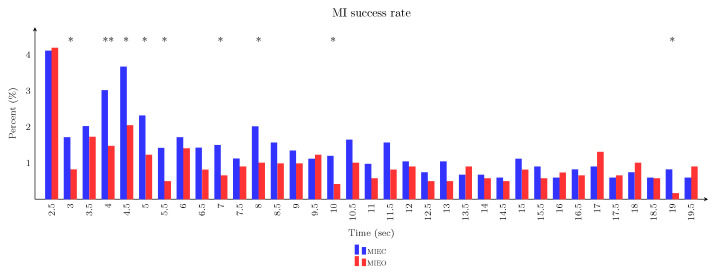
The *blue* and *red* bars represent the MI task success rate in MIEC and MIEO conditions, respectively. The statistically significant differences in MI performance across each time bin are indicated (${}^{*}$: p<0.05, ${}^{**}$: p<0.01).

In a successful attempt, the external robotic device coupled with the designed hybrid BCI was triggered by the patient’s imagery of the right (affected) hand, either with closed- or open eyes. For each eye condition, we assessed the number of successful MI attempts and the duration of each successful MI process as the behavioural performance index. A Chi-Square test was performed to determine whether the proportion of MI duration (the time between imagery onset and triggering the robotic splint) in each histogram bin that reflected a reaction time index, was equal between both different eye conditions. In successful trials, the minimum and maximum MI times were 2.5 and 19.5 seconds, respectively. Whereas, in unsuccessful trials in which the patient failed to hit the robotic splint, the MI time was 20 seconds. The percentage of unsuccessful trials was approximately 52% and 66% for the MIEC and MIEO conditions. There was a significant difference between these two conditions ($\chi^{2}=50.24$, p<0.001) revealing that the patient was more likely to complete the MI task when he had to keep his eyes closed. In addition, in successful trials, as Figure 7 shows, the patient was more likely to complete the MI task with the longer imagery time when he had to keep his eyes open. In successful trials, the average time for triggering the robotic splint took 8.6 ± 0.52 (mean ± SEM) and 9.2 ± 0.69 seconds for the MIEC and MIEO conditions, respectively.

## 4. Discussion

### 4.1. Summary

We used EEG recordings of a stroke patient during longitudinal MI BCI training to assess how cortical sensorimotor oscillations might be modulated by manipulating eye conditions while he imagined moving the affected hand. To this end, we sought to propose a comprehensive mathematical framework based on multiway tensor decomposition to extensively study narrow-band sensorimotor EEG rhythm interactions during MI with EO and EC. In this approach, the multi-channel EEG signal was modelled separately by the three-way PARAFAC to obtain unique time-space-spectral signatures for each training day and eye condition. Individual spatial and spectral characteristics of the identified signatures were then averaged to create a general representation of the most dominant cortical motor-related sources that are active during the MI task. By projecting obtained general weights to the log-transformed oscillatory part of the EEG spectrum recorded, we reliably identified a set of time scores related to these spatial and frequency signatures. Finally, non-parametric, cluster-based permutation statistics were applied to the average of those time scores in order to reveal differential dynamics in sensorimotor modulations with EC and EO during different MI time frames, including movement preparation, movement initiation, and movement completion.

### 4.2. Main Findings

We found that the oscillatory neural activity during MIEC and MIEO was modulated differently, providing additional evidence that closed-eyes and open-eyes are fundamentally different behaviours [20,29]. When MIEC trials were compared with MIEO trials, three key narrow-band rhythms were identified with differently modulated oscillatory dynamics: (I) Bilateral 15.5 Hz atom in the movement preparation phase, (II) Contralateral 8.0 Hz and 11.5 Hz atoms in the movement initiation phase, and (III) Contralateral 11.5 Hz atom in the movement completion phase. Furthermore, the dynamic of the ipsilateral 9.5 Hz oscillatory atom differed between the eye conditions in the movement preparation and movement initiation phases. The sensorimotor oscillatory dynamic of the contralateral 14.0 Hz atom was also quantitatively different during the movement completion phase. In other cases, although there were some statistically significant clusters between the eye conditions’ modulatory behaviour, the modulations evoked during different phases of performing MI showed similar dynamic patterns across narrow-band sensorimotor oscillatory rhythms.

### 4.3. Physiological Interpretation of the Findings

First of all, it should be stressed that, despite the similarities or dissimilarities between the results presented in the current study and those outlined below, we cannot directly compare the result of the current study with the previous research. The previously published results are based on relatively wide-band frequency ranges in the raw EEG spectrum, which may combine multiple oscillatory peaks at a particular spatial position [41]. These are mainly univariate approaches suffering low spatial sensitivity and cannot leverage the volume conduction effect [63]. Furthermore, they are not generally specific to individuals and do not take into account the differences in spatial and spectral characteristics among subjects [36,37]. Meanwhile, these neural characteristics in stroke patients are influenced by lesion heterogeneity and a degree of functional impairments [31,64], therefore subject-specific analytical frameworks need to be taken into consideration, even in cohorts of subjects.

The suppression of mu rhythm has traditionally been thought to be primarily an index of human mirror neuron system activity when subjects engage in a motor-related task [65,66,67]. However, this suppression may not be considered a reliable indicator of activation in the mirror neuron system given that mu rhythm can be easily mixed with alpha rhythm as they fall within the same frequency range in the canonical EEG frequency definition [68,69]. While suppression of the mu rhythm is not dependent on eye condition, alpha activities are blocked when the eyes are opened [70,71].

This has largely been confirmed in our study, where similar EC and EO oscillatory 8.0 Hz dynamics were observed during the different phases of MI. By contrast, opening the eyes blocked 9.5 Hz desynchronization and changed its dynamic patterns, while 9.5 Hz modulations in the EC condition still represented significant desynchronization relative to the baseline (Figure 6 and Figure 5, right column). Therefore, 8.0 Hz and 9.5 Hz oscillatory atoms can be considered as mu and alpha rhythms, as has been demonstrated in an earlier study [41].

The stronger contralateral 8.0 Hz desynchronization underlying MIEO (Figure 6, left column) can be interpreted as a mechanism reflecting functional neuronal inhibition in the task-irrelevant brain region. This finding supports the theory that oscillations in the canonical alpha band reflect gating by inhibition, revealing that alpha activity decreases in task-relevant regions and increases in task-irrelevant regions [72]. Haegens et al. found that the alpha-band activity increased in the sensorimotor cortex ipsilateral to the engaged hand in a somatosensory working memory task, while it decreased in the contralateral hemisphere [73]. Furthermore, the stronger 8.0 Hz neuronal inhibition (greater desynchronisation) in MIEC condition than that of MIEO in both ipsi- and contralateral cortices (see Figure 6) suggests an interruption in attentional processing caused by opening the eyes [74]. The pre-movement 9.5 Hz rhythm desynchronization observed in the MIEC condition (Figure 5) may be explained by the notion that pre-stimulus alpha oscillatory activity modulates the subsequent processing of visual and somatosensory stimuli [72]. In line with this finding, in a visuomotor task, global decreases of canonical alpha synchronization in the posterior and sensorimotor regions were observed a few seconds before the action [75].

Evidence from previous neuroimaging recordings in humans [76] and monkeys [77] has demonstrated sensorimotor beta desynchronization after stimulation of a movement followed by synchronization after movement termination. Furthermore, the relative increase of beta power in the movement preparation phase has been found to be associated with the anticipatory processes when a task-relevant sensory cue is expected [76,78,79]. Consistent with this notion, we observed similar dynamic patterns in the modulations 14.0 Hz (ipsilateral), 15.5 Hz, 17.5 Hz oscillatory atoms (Figure 5 and Figure A1), which are within the range of canonical beta.

Recent studies have also identified a distinct role for sensorimotor rhythmic activities in the human motor cortex, in which the ipsilateral alpha-band oscillatory power was increased during the stimulation of a movement, whereas the contralateral beta-band oscillatory power was concurrently decreased [80,81]. Here, we have provided additional evidence for the existence of such dissociation of lower and higher sensorimotor rhythms in the motor imagery process. Our results showed that a bilateral decrease of 9.5 Hz oscillatory power (predominantly in the ipsilateral cortex) was accompanied by increases of 15.5 Hz (predominantly in the contralateral cortex) and 17.5 Hz oscillatory power in the MPT phase. In addition, a bilateral increase of 11.5 Hz oscillatory power (predominantly in the contralateral cortex) was accompanied by decreases of ipsilateral 15.5 and 17.5 Hz oscillatory power in the MIT phase. Moreover, the increase of contralateral 11.5 Hz oscillatory power was accompanied by the decrease of ipsilateral 15.5 Hz power in the MCT phase. However, the laterality of the lower and higher sensorimotor modulations in our study is contrary to those of Brinkman et al. [80] and Stolk et al. [81]. This inconsistency is likely to be related to the fact that the left hemisphere of the stroke survivor who participated in our study showed ischemia extending from the frontotemporal to parietal areas [41]. This may have affected cortical neural generators in the sensorimotor cortex contralateral to the impaired hand. These results were also more obvious for the dynamic modulations underlying MI with EC compared to EO.

### 4.4. Technical Considerations in Tensor Construction

In tensor analysis, identifying the optimum number of factors as a crucial parameter that might severely impact the decomposition quality, is still under debate. A recent study comparing factor number selection methods for PARAFAC decomposition in simulated EEG signals [57] revealed that commonly used methods such as the proportion of variance explained [82] and core consistency diagnostics (CORCONDIA) [83] are not appropriate when the data follows a non-negative structure, which is the case in the current study. In the simulated EEG data [57], the best results were obtained by automatic relevance determination (ARD) [84] and cumulative component clustering (tripleC) [57] algorithms. The tripleC is an approach based on cluster analysis of a merged set of factors from many PARAFAC models with a different number of factors, which has been successfully applied in our other studies [46,47]. In the present study, according to the tripleC method, we computed 15 separate PARAFAC models in which the number of factors ranged from 6 to 20. We were aware from the previous mirror-box data analysis [41] that at least five different narrow-band oscillatory rhythms were present in the patient’s analyzed data tensor (named as theta, mu, alpha, smr, beta). Moreover, these rhythms may occur in several versions for example, smr 1/smr 2 or beta 1/beta 2/beta 3 [41]. Therefore, we started with 6 factors as the minimum number of components in the PARAFAC model. Then, to ensure the presence of all detected narrow-band rhythm lateralization [34,35], we significantly increased the number of factors to 20. Due to numerical and computation issues, we did not consider higher values. Despite a PARAFAC model with *K* factors not being fully inherent in a PARAFAC model with K+1 factors, we hypothesise that dominant and stable (with low variance) signatures (frequency and spatial) would be present in the majority of models. In the second step, estimated factors from all 15 PARAFAC models were merged into one set, and a cluster analysis was applied. Because we do not know a priori the number of dominant/stable factors, clustering techniques requiring the number of clusters as input are not suitable for our case. Therefore, we focus on a non-parametric, density-based clustering approach as implemented in the DBScan [58] algorithm which can detect an optimal number of clusters based on the data structure. Finally, the representatives of the most dominant clusters (clusters with many factors) were considered subject-specific factors.

Here, we focused primarily on the oscillatory motor-related EEG rhythms. Neural oscillations co-existing with ubiquitously present non-oscillatory activity are thought to be generated by distinct neural mechanisms [85,86]. Therefore, it is essential to disambiguate ongoing brain activities from non-oscillatory components to increase spectral precision and physiological interpretability when studying task-related spectral modulations over brain rhythms [81,87]. By separating the non-oscillatory part from the raw EEG spectrum using the IRASA technique, we obtained three congruent frequency clusters representing the significant difference between the eye conditions (Figure 3). In addition, considering spectral features of intracranial recordings from the human cortical surface during the performance of an MI task revealed uncorrelated alpha and beta oscillatory activity in the sensorimotor cortex, while, using the original power spectra (without the separation of oscillatory and non-oscillatory activity in the power-spectrum), those rhythms were temporally and spatially correlated [81]. We believe that considering the oscillatory part of neural oscillations in the tensor construction is a precursor to studying the dynamics of the underlying EC and EO neuronal population more accurately.

### 4.5. Implications for BCI Domain

Behavioural analysis showed that task performance was affected by eye conditions during the MI process. In our study, the subject performed significantly more successful MI efforts with less MI time during the course of rehabilitation while his eyes were closed. This result supports a recent study showing that while performing a voluntary isometric flexion task, a significant behavioural improvement was observed when subjects had to keep their eyes closed [29]. Moreover, performing a motor task with closed eyes with haptic or auditory feedback could improve concentration and attention during MI BCI tasks [88]. This issue comes to the fore in clinical settings in particular, given that BCI control paradigms based on visual input require a high level of attention and visual focus that impose additional difficulties and fatigue in post-brain injury patients. This in turn could reduce the efficiency of rehabilitation and slow down neuroplasticity mechanisms. On the other hand, a BCI system that relies on visual input is prone to producing eye-blinking artefacts or saccadic eye movements leading to a low signal-to-noise ratio and imposing precise pre-processing steps. This finding, while preliminary, could have further implications for developing alternative, more efficient BCI paradigms enabling patients to self-regulate their specific neural substrates, as described above, during MI with EC to drive a matched sensory feedback coupled to a BCI system.

## 5. Conclusions

This study provides empirical evidence that neural oscillations in the sensorimotor cortices are differentially modulated by the simulation of movements with different eye conditions. The use of PARAFAC enables the simplification of the sophisticated longitudinal EEG-BCI data structure by reducing its dimensionality and providing a compact, informative, and physiologically interpretable framework. Despite the highly promising findings of this study, it is necessary to replicate the study on a larger pool of subjects containing both stroke patients and healthy subjects. However, our findings pave the way for the use of multidimensional signal processing frameworks to enable further research into latent mechanisms underlying movement neurophysiology and refine the current outlook on the human sensorimotor system.

## Data Availability

The data supporting the results of this research will be available by the authors upon a reasonable request.
